# Scents of Adolescence: The Maturation of the Olfactory Phenotype in a Free-Ranging Mammal

**DOI:** 10.1371/journal.pone.0021162

**Published:** 2011-06-27

**Authors:** Barbara A. Caspers, Frank C. Schroeder, Stephan Franke, Christian C. Voigt

**Affiliations:** 1 Department of Animal Behaviour, University of Bielefeld, Bielefeld, Germany; 2 Boyce Thompson Institute and Cornell University, Ithaca, New York, United States of America; 3 Institute of Organic Chemistry, University of Hamburg, Hamburg, Germany; 4 Evolutionary Ecology Research Group, Leibniz Institute for Zoo and Wildlife Research, Berlin, Germany; University of Queensland, Australia

## Abstract

Olfaction is an important sensory modality for mate recognition in many mammal species. Odorants provide information about the health status, genotype, dominance status and/or reproductive status. How and when odor profiles change during sexual maturation is, however often unclear, particularly in free-ranging mammals. Here, we investigated whether the wing sac odorant of male greater sac-winged bats (*Saccopteryx bilineata*, Emballonuridae) differs between young and adults, and thus offers information about sexual maturity to potential mating partners. Using gas chromatography – mass spectrometry, we found differences in the odorants of young and adult males prior and during, but not after the mating period. The wing sac odorant of adult males consists of several substances, such as Pyrocoll, 2,6,10-trimethyl-3-oxo-6,10-dodecadienolide, and a so far unidentified substance; all being absent in the odor profiles of juveniles prior to the mating season. During the mating season, these substances are present in most of the juvenile odorants, but still at lower quantities compared to the wing sac odorants of adults. These results suggest that the wing sac odorant of males encodes information about age and/or sexual maturity. Although female *S. bilineata* start to reproduce at the age of half a year, most males of the same age postpone the sexual maturation of their olfactory phenotype until after the first mating season.

## Introduction

Maturing animals do not only develop full reproductive performance, but also converge towards an adult phenotype. This process of maturation extends well beyond morphological and physiological changes [1], and also includes the olfactory phenotype [2]. For example, many sexually selected scent glands are regulated by plasma androgen levels, and when individuals maturate, external glands may become activated and thus secret volatiles in response to elevated plasma androgen levels [3]–[6]. Age differences in odor profiles have been documented for a variety of mammals such as laboratory rats: [7]–[8], mandrills: [9] and Asian elephants: [10]. But how and when odor profiles change during sexual maturation remains often unclear, particularly in free-ranging mammals. We asked whether odorants of a free-ranging mammal encodes for information about sexual maturity, and if so, which volatiles are changing during sexual maturation.

Although bats are mostly known for their acoustical capabilities, they also possess a variety of scent glands (e.g. [11]–[12]) whose secretions are predominantly used in social contexts [13]–[17]. Here, we investigated the ontogeny of a chemical signal that is used for courtship displays in adult male greater sac winged bats, *Saccopteryx bilineata*, and, thus, most likely shaped by sexual selection. Males of the Neotropical greater sac-winged bat, *Saccopteryx bilineata,* possess a pouch in the wing membrane which is filled with an odoriferous liquid [18]–[19]. The wing pouches are free of any glandular tissue [18] and male greater sac-winged bats spend up to one hour each day cleaning the pouches and mixing the odorants from saliva, urine and gland secretion [19]. Past studies have confirmed that, apart from acoustic and visual signals, courtship behavior of males includes predominantly olfactory signals [20]. In addition, wing sac odorants of male *S. bilineata* represent a composite signal which encodes information about species [16], individual identity and season [21].

*Saccopteryx bilineata* has an annual reproductive cycle with a short mating season in early December [22]–[23]. Usually, females give birth to a single young in May or June. Reproductive success of sexually mature juveniles differs largely between males and females of the same age cohort (cohort members  =  CM). Whereas almost all female CM get pregnant in their first mating season at an age of about 6 months, only approximately 5% of male CM sire offspring at this age [24], indicating that, in contrast to females, most male juveniles do not reach sexual maturity at this early age.

We used gas chromatography and mass spectrometry to find out whether the chemical signal used by male greater sac-winged bats might encode information about age and/or sexual maturation. In particular, we analyzed the wing sac odorant of juvenile and adult males and asked whether the composition of wing sac odorants differ between juveniles and adults.

### Results

**Figure 1:**
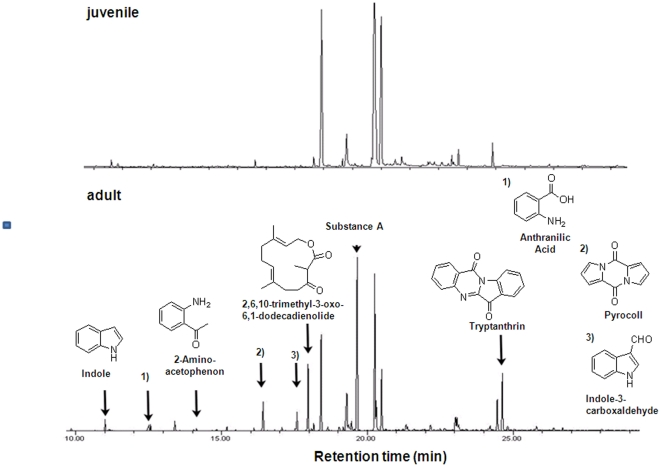
Example chromatograms of a juvenile and adult male *S. bilineata* and the structures of the male specific substances.

**Figure 2:**
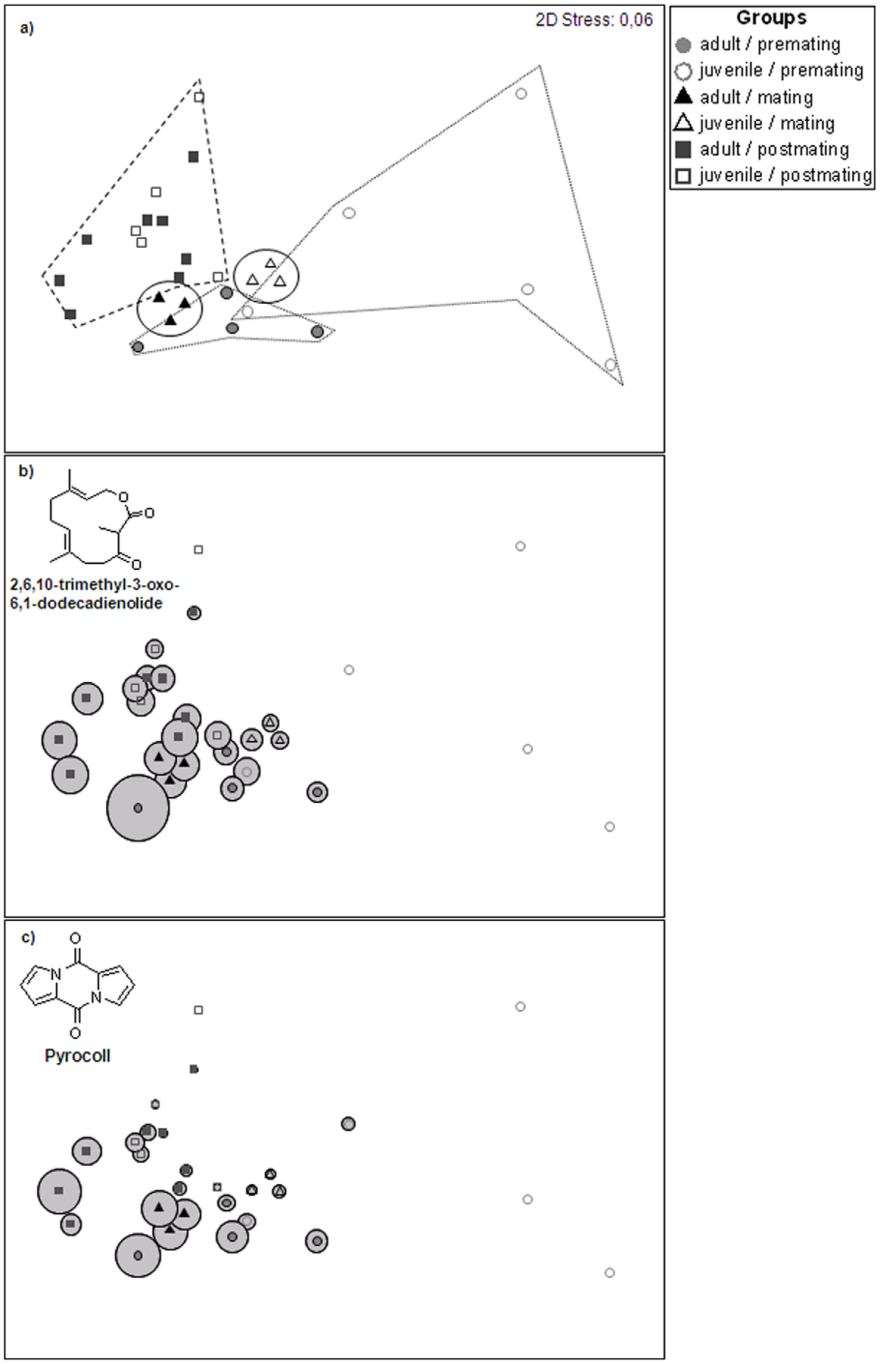
Weekly odor sample compositions and the relative amounts of two male specific substances exemplarily. For reasons of graphic power, we averaged the samples over individual, age and week. The graph shows the weekly odor sample compositions (a) of juvenile (open symbols) and adult (closed symbols) male *S. bilineata* prior (circles), during (triangles), and after the mating season (squares) and the relative amounts of two male specific substances (b,c). The two-dimensional non-metric Multidimensional Scaling (nMDS) plot of odorant composition is based on the Bray-Curtis Similarity indices, i.e. the closer the samples appear on the plot, the more similar the samples are. The bigger the bubble is, the higher the relative amount of the specific substance. The orientation in a nMDS is arbitrariness and axes are dimensionless.

The composition of wing sac odorants differed between adult and juvenile male *S. bilineata* (Figure 1) prior (Juvenile  =  Juv: N = 5; Adult  =  Ad: N = 5; *ANOSIM* (age as factor): *Global R* = 0.32; *p* = 0.022) and during (Juv: N = 8; Ad: N = 16 *ANOSIM*: *Global R* = 0.37; *p* = 0.007), but not after the mating season (Juv: N = 6; Ad: N = 9 *ANOSIM: Global R* = 0.033; *p* = 0.3, see Figure 2a).

Age differences prior the mating season were mainly due to the absence of male specific substances, especially substance A and 2,6,10-trimethyl-3-oxo-6,10-dodecadienolide (Table 1, Figure 2b). These substances were only present in one out of five juvenile odorants (20%) prior the mating season. During the mati

**Table 1:** Major substances responsible for dissimilarity between adult and juvenile male *S. bilineata* during the premating season.

Premating season	AdultsAv. Abundance (%)	JuvenilesAv. Abundance (%)	Contribution (%) to Dissimilarity	Cumulative contribution to Dissimilarity (%)
Substance A ♦	2.24	0.51	18.06	18.06
2,6,10-trimethyl-3-oxo-6,10-dodecadienolide♦	1.89	0.32	16.83	34.88
Cholesterol	2.46	2.3	13.04	47.92
Tryptanthrin ♦	1.77	0.66	12.20	60.13
Hexadecanoic Acid	3.41	3.65	8.97	69.10

Substances are ordered in decreasing contribution (SIMPER). Av. =  Average; Cum.  =  Cumulative; Contr.  =  Contribution, ♦ =  male specific substance.

ng season, most of the juvenile odorants (five out of eight individuals; 63%) contained male specific substances, but at lower relative quantities compared with the odor profiles of adult males (Table 2; Figure b,c). For odor profiles collected from males after the mating season, we found neither differences in the presence of substances nor in the amount of substances between the odorant of juveniles and adults.

**Table 2:** Major substances responsible for dissimilarity between adult and juvenile male *S. bilineata* during the mating season.

Mating season	AdultsAv. Abundance (%)	JuvenilesAv. Abundance (%)	Contribution (%) to Dissimilarity	Cum. Contrib. to Dissim. (%)
Substance A ♦	2.70	1.16	18.51	18.51
Pyrocoll ♦	1.53	0.39	12.43	30.95
2,6,10-trimethyl–3-oxo-6,10-dodecadienolide ♦	1.76	0.77	11.60	42.54
Octadecanoic Acid	1.97	2.78	9.23	51.77
Tryptanthrin ♦	2.08	1.51	9.18	60.95
Anthranilic Acid	0.71	0.56	6.68	67.64

Substances are ordered in decreasing contribution (SIMPER). Av. =  Average; Cum.  =  Cumulative; Contr.  =  Contribution, ♦ =  male specific substance.

## Discussion

During ontogeny, animals mature until they have reached the adult phenotype. While the process itself is widely recognized due to the accompanying morphological and physiological changes, the maturation of the olfactory phenotype remains largely unknown for most animals. Here we show that juvenile and adult male *Saccopteryx bilineata* differed in the composition of the wing sac odorants prior and during the mating period but not afterwards. In addition, we demonstrate that prior to the mating season, the majority of juvenile wing sac extracts lacked the male specific substances 2,6,10-trimethyl–3-oxo-6,10-dodecadienolide, substance A and Pyrocoll (Figure 2b,c).

During the mating season, these substances were present in most of the samples, however at smaller quantities. Two of these substances, namely 2,6,10-trimethyl-3-oxo-6,10-dodecadienolide and substance A are species specific [16]. Since the presence of these compounds coincides with the time of sexual maturity, we infer that some, if not all of these substances may serve as cues for sexual maturity because they may be associated with androgen regulated glands. Testosterone levels are indeed higher in adult male *Saccopteryx* than in juvenile males that remain with their mother until their first mating season [25]. In male *S. bilineata*, three likely candidates for scent glands are found in the facial region [17]. These facial glands, particularly those of the mandibular region, contribute to the male wing sac odorant [17], [19], and we speculate that these glands may be androgen regulated like many sexually dimorphic glands of other mammalian species [4].

In laboratory rats, it has already been demonstrated that sexual maturity is signaled by the presence of a few specific substances [9]. Osada and co-workers [9] showed that the urine composition of adult and prepubescent males differ significantly, due to the presence of specific volatiles. Additional behavioral experiments showed that urine containing these specific substances is more attractive to adult females, indicating that the composition of urine in male rats is used by females to gain information about sexual maturity.

Our behavioral observations and odor analyses suggest that the age at which male *S. bilineata* change their olfactory phenotype from adolescence to adulthood varies among individuals. With a few exceptions, the majority of male juveniles do not reach the olfactory phenotype during their first mating season. In most mammals, timing of maturation is influenced by intrinsic factors such as age, body size, or indirectly by body condition which is linked to environmental factors [26]–[27]. In some species, trade-offs occur between fitness-relevant components such as maturation and reproductive performance [28]. Early maturation might ultimately result in early reproduction and thus in short term reproductive success, but in lower lifetime reproductive success, because of lower competitive ability [29]. In the current study, two juvenile males took over a harem territory within their first year. Both males showed an adult like odor profile and one of the two even sired an offspring (Nagy pers. communication). These findings are consistent with an earlier study, which reported that approximately 5% of males sired offspring at the age of 6 month [24]. Thus, only a small proportion of juvenile males reaches maturity and gains reproductive success during the first mating season. However, if reproduction is unlikely for juvenile males, it might be evolutionary stable to postpone reproduction until the likelihood of reproduction increases. Another explanation for the lack of adult odor profiles in juvenile males is that juveniles may also mask their gender to avoid excessive male-male competition during their first months in the natal colony.

Delayed maturation of morphological traits is a well documented phenomenon in birds [30]–[31]. In lazuli buntings *(Passerina amoena)* for example, male yearlings show a high variation of plumage coloration and males with a dull, female like plumage coloration or males with a bright, adult male like plumage coloration have a higher reproductive success than intermediate plumage colored yearlings [31].

Delayed maturation as a mechanism to avoid inbreeding [32] can probably be excluded, since greater sac-winged bats have female dispersal and the only close relative females in the colony are the mothers, which are still present the next mating season. It might be more likely, although at the moment very speculative that harem males suppress reproduction of yearlings to avoid competition, especially since most juveniles within the harem are sired by other males [20], [24].

Whether juveniles lacking the adult odorant are sexually immature or whether they merely avoid smelling like adults, i.e. follow a strategy of cryptic behavior, cannot be concluded with certainty from our data. But since the majority of juveniles do not sire offspring and have a distinct odor profile from adults, we assume that the presence of adult odorants coincides with sexual maturation and that females gain information about sexual maturity based on the wing sac odor profile. Olfactory cues are often by-products of every day metabolic processes and the process of sexual maturation in mammals is generally characterized by hormonal changes, leading to the assumption that chemical cues are probably difficult to manipulate and thus might be honest signals of sexual maturity.

## Materials and Methods

### Ethics Statement

Bat capturing and odor sample collection complied with the current laws of Costa Rica (Minesterio de Ambiente y Energia (Permission number: 135-2004-OFAU)). We captured the bats using nylon mist nets (Avinet, Dryden, USA, CH2, Mist Net) and collected odor samples directly at the site of capture. For odor collection, we wiped out the wing pouches with a piece of cotton wool. After odor collection the bats were released.

### Study Site and Sample Collection

This study was conducted in the vicinity of “La Selva” Biological Station (10°25′ N. 84°00′ W) in Costa Rica (Province Heredia) administered by the Organization of Tropical Studies. We collected samples from September 2003 until January 2004, and November 2004 until March 2005 from four different colonies. We refer to the mating season as the period between the 50^th^ and 52^nd^ calendar week according to Voigt and co-workers [22]–[23], [25]. Samples collected prior to this period belonged to the pre-mating period and samples collected after this period belonged to the post-mating period. Since study colonies were part of a long-term project on the socio-biology of *Saccopteryx bilineata*, all colony members have been individually marked with colored plastic bands (AC Hughes LTD., Middlesex, UK, size XCL).

We captured bats between 0500 and 0600 hours when bats returned to the daytime roost or in the roost after their arrival (0600–0830 hours). For our study period, we categorized bats as subadults when they were born during the same year in which they were captured and all others as adults (older than 1 year). For a few individuals with unknown birth date, we assessed the age by the color and the structure of their wing sacs [33]. In all cases, we collected as much liquid as possible by wiping out the wing sac with an odor free piece of cotton (Hartmann, Heidenheim, Germany, DIN 61640-CO, 100% cotton). For this, we twirled approximately 5 mg of cotton to a dapper (∼1 cm length, 0.3 cm width) and stored them in a Teflon-capped glass vial (2 ml, Rotilabo®, Karlsruhe, Germany). We washed and dried the cotton as well as the glass vial with dichloromethane (99.9%) before taking odor samples. All samples were stored in a freezer at –20°C until analysis. Each time we took a subsequent sample from the same individual, we altered between the left and right wing sac (i.e. right wing, left wing, right wing etc.) to guarantee that the odor profiles were not influenced by the previous sampling event. We added 100 µl of chemically pure dichloromethane (99.9%) to all samples for preservation. After odor collection, we released all bats at the site of capture.

### Chemical Analyses

We included 77 samples from 32 males in our study. All samples were analyzed by gas-chromatography and mass spectrometry (GC-MS) using a Hewlett-Packard 5890 GC equipped with a 30 m J&W (J&W Scientific, Folsom, CA) DB5-coated capillary column linked to a Hewlett-Packard mass selective detector (MSD; 70eV EI). Odor samples were extracted by squeezing out the cotton dapper using a blunt point syringe (50 µl, Hamilton®, Bonaduz, Switzerland). We transferred the extracts each into glass vials (2 ml, Rotilabo®, Karlsruhe, Germany), that were equipped with a 100 µl inlet (Rotilabo® , Karlsruhe, Germany). The extracts were concentrated by evaporation to approximately 5 µl before analysis. Data were collected under the following GC conditions: 1.5 µl split-less injection, chemically pure helium as the carrier, 60°C inlet temperature, 3 min initial time, 10°C/min rate, 280°C final temperature, 20 min final time. Mass spectra were analyzed and compared to library spectra or in case of species specific substances by spectra of authentic samples to identify substances and to determine the composition of wing sac odor samples. One male specific substance, indicated as substance A, has not yet been defined.

We quantified the variation of wing sac odor composition by analyzing 12 focus substances, which met one of the following criteria: they were either found to be male-specific, represented a fatty acid, or were identified as a steroid. In order to determine the male-specific substances, we compared wing sac odorants of adult males with odorants collected from the rudimental wing sacs of females. In addition to male specific substances, we used fatty acids and steroids for the analysis of odor maturation, because previous studies have already shown their biological relevance in social and sexual contexts of other mammal species [34]. In all GC runs, we quantified the relative contribution (%) of peak area of each focal substance to the total peak area of all focal substances. This procedure guaranteed that all samples were comparable, since the total amount of wing sac odorant differed among individuals and among samples of the same individual.

### Statistical Analyses

We compared the odor composition by computing a similarity matrix based on the Bray-Curtis-Similarity Index [35] and analyzed potential differences between a priori defined groups (factor: age) using a non parametric analysis of similarities (ANOSIM, [36]). We averaged samples per individual in case of more than one sample per individual within a season group. In case of group differences, we ran the SIMPER routine to determine the discriminating variables. Statistical analyses were performed using PRIMER 6.1.12 (Primer-236 E 2000 Ltd., Plymouth, UK). The significance level was set to α = 0.05 and we used two-tailed tests.
